# Roles of GDF-15 in Acute Heart Failure: from Mechanistic Insights to Clinical Applications

**DOI:** 10.7150/ijms.138771

**Published:** 2026-08-22

**Authors:** Paisit Kosum, Nonthikorn Theerasuwipakorn, Siriporn C. Chattipakorn, Nipon Chattipakorn

**Affiliations:** 1 Division of Cardiovascular Medicine, Department of Medicine, Faculty of Medicine, Naresuan University, Phitsanulok, Thailand; 2 Cardiac Center, Naresuan University Hospital, Phitsanulok, Thailand; 3 Division of Cardiovascular Medicine, Department of Medicine, Faculty of Medicine, Chulalongkorn University, King Chulalongkorn Memorial Hospital, Bangkok, Thailand; 4 Cardiac Center, King Chulalongkorn Memorial Hospital, Bangkok, Thailand; 5 Cardiac Electrophysiology Research and Training Center, Faculty of Medicine, Chiang Mai University, Chiang Mai, Thailand; 6 Center of Excellence in Cardiac Electrophysiology Research, Chiang Mai University, Chiang Mai, Thailand; 7 Department of Oral Biology and Diagnostic Sciences, Faculty of Dentistry, Chiang Mai University, Chiang Mai, Thailand; 8 Cardiac Electrophysiology Unit, Department of Physiology, Faculty of Medicine, Chiang Mai University, Chiang Mai, Thailand; 9 The Academy of Science, The Royal Society of Thailand, Bangkok, Thailand

**Keywords:** Acute heart failure, GDF-15, biomarkers, cardiomyocyte stress, risk stratification

## Abstract

Acute heart failure (AHF) is a clinical syndrome characterized by the rapid or gradual onset of symptoms and signs of heart failure that are severe enough to require urgent evaluation and treatment. It may represent *de novo* heart failure or acute decompensation of pre-existing chronic heart failure. Natriuretic peptides remain the cornerstone biomarkers for diagnosis and risk assessment, but predominantly reflect hemodynamic stress and incompletely capture inflammation, oxidative injury, cellular damage, and multiorgan dysfunction. Growth differentiation factor-15 (GDF-15), a stress-responsive cytokine of the transforming growth factor-beta superfamily, may complement natriuretic peptides by reflecting these non-hemodynamic pathways. We conducted a targeted PubMed search from database inception to January 2025 and identified 16 original mechanistic and clinical studies relevant to GDF-15 in AHF. In cultured cardiomyocytes, biomechanical stretch increased GDF-15 expression approximately 24.8-fold, while recombinant GDF-15 reduced ischemia/reperfusion-related necrosis and apoptosis through phosphoinositide 3-kinase/Akt signaling and attenuated agonist-induced hypertrophy through SMAD2/3-associated pathways. In animal models, GDF-15 deficiency increased infarct size and cardiomyocyte apoptosis after ischemia/reperfusion and produced greater hypertrophy and an 84% reduction in contractile performance after 2 weeks of pressure overload, compared with a 25% reduction in wild-type mice. Clinical studies generally showed that higher admission or discharge GDF-15 concentrations and increasing serial levels were associated with mortality or heart-failure rehospitalization. For example, serial GDF-15 remained independently associated with adverse outcomes after simultaneous modelling with established biomarkers (adjusted hazard ratio 1.44 per 1-SD increase in log GDF-15), and a multimarker model incorporating GDF-15, NT-proBNP, and troponin I achieved an area under the curve of 0.785. GDF-15 is not currently recommended for routine heart-failure management since assays and thresholds are not standardized and no prospective trial has shown that GDF-15-guided treatment improves outcomes. Its most plausible near-term role is therefore as one component of a multimarker risk-stratification strategy.

## Introduction

Acute heart failure (AHF) is defined as the rapid or gradual onset of symptoms and signs of heart failure that are sufficiently severe to require urgent medical evaluation and treatment, commonly resulting in hospitalization. AHF includes both *de novo* heart failure and acute decompensation of pre-existing chronic heart failure; the latter is often termed acute decompensated heart failure. Since the present review focuses on the acute clinical presentation rather than chronic stable heart failure, the term AHF is used consistently throughout. AHF remains a major cause of hospitalization and is associated with substantial early mortality and recurrent admission after discharge 1-3. Clinical assessment together with B-type natriuretic peptide (BNP) or N-terminal pro-B-type natriuretic peptide (NT-proBNP) is central to diagnosis and risk assessment. However, natriuretic peptides predominantly reflect myocardial wall stress and do not fully characterize inflammation, cellular injury, neurohumoral activation, oxidative stress, or multiorgan dysfunction 1, 2, 4, 5.

These limitations have stimulated interest in biomarkers that reflect non-hemodynamic pathways. Growth differentiation factor-15 (GDF-15), a stress-responsive member of the transforming growth factor-beta (TGF-beta) superfamily, is expressed at low levels in most healthy tissues but is strongly induced by ischemia, oxidative and nitrosative stress, inflammation, and mechanical strain 6-8. Cardiomyocytes, endothelial cells, activated macrophages, and several extracardiac tissues can produce GDF-15 during tissue injury 7, 9-16. Circulating GDF-15 concentrations are associated with adverse outcomes in both chronic and acute heart failure, although the biomarker is not cardiac-specific 7, 17-23.

Experimental studies have identified several pathways through which GDF-15 may influence the myocardial response to stress. In neonatal rat ventricular cardiomyocytes (NRVCMs), biaxial stretch to 112% of baseline length for 24 hours increased GDF-15 expression approximately 24.8-fold; this response was reduced by 65.9% with angiotensin II type 1 receptor blockade 8. During simulated ischemia/reperfusion, GDF-15 induction depended on nitric oxide-peroxynitrite signaling, whereas recombinant GDF-15 reduced necrosis and apoptosis through phosphoinositide 3-kinase (PI3K)/Akt signaling 11. In a separate model, GDF-15 attenuated agonist-induced hypertrophy and activated SMAD2/3, Akt, and ERK1/2 signaling 6. Consistent with these cellular findings, GDF-15-deficient mice developed larger infarcts after ischemia/reperfusion and greater hypertrophy and ventricular dysfunction after pressure overload 6, 11.

Clinical studies have evaluated GDF-15 at admission, discharge, and during follow-up. Although findings differ according to population, assay, sampling time, and outcome definition, most studies report that persistently high or increasing GDF-15 concentrations identify patients at greater risk of mortality or heart-failure rehospitalization 18-25. Importantly, GDF-15 has frequently retained prognostic value after adjustment for NT-proBNP and clinical risk factors, supporting its potential role within, rather than as a replacement for, multimarker risk assessment 19, 20, 22, 23, 26.

This narrative review synthesizes experimental and clinical evidence regarding GDF-15 in AHF, identifies limitations in the current literature, and proposes priorities for clinical translation. PubMed was searched from database inception to January 2025 using the terms “GDF-15” AND “acute heart failure.” Original studies were eligible if they examined GDF-15 expression, regulation, biological effects, or prognostic value in an experimental model relevant to cardiac injury or in patients with AHF or a recent heart-failure hospitalization. Sixteen core original studies were included in the structured evidence tables; additional publications were used to provide biological and guideline context.

## Biomarkers in Heart Failure

Biomarkers serve as essential diagnostic tools for heart failure (HF), helping to evaluate patient risk and manage their conditions. The complex pathophysiology of heart failure, which includes hemodynamic stress, neurohormonal activation, inflammation, oxidative stress, and multiorgan dysfunction, prevents the use of any single biomarker. Thus, the current approach to biomarker assessment utilizes multiple markers to enhance both predictive power and therapeutic decision-making 4, 5.

BNP and NT-proBNP serve as the most validated biomarkers used for diagnosis and predicting outcomes in heart failure 1. The levels of BNP and NT-proBNP serve as indicators of myocardial wall stress while also predicting both the severity of heart failure and treatment response and short-term clinical results 1, 2, 4, 26. Other widely used biomarkers include cardiac troponins (cTnI, cTnT), which serve as indicators of myocardial injury and also predict poor outcomes in the absence of acute coronary syndrome 20, 22, 27, soluble ST2 (sST2) which reflects myocardial fibrosis and remodeling 28-30, Galectin-3 which is associated with inflammation and fibrosis 20, 22, 31, and Cystatin C which indicates renal dysfunction, a decisive prognostic factor in HF 20, 32, 33.

## Emerging and Complementary Biomarkers

Several emerging relevant biomarkers have also been reported. These include GDF-15, sST2, Galectin-3, and cystatin C. GDF-15 functions as a stress-responsive cytokine, indicating inflammation, oxidative injury, and tissue damage. Copeptin functions as a surrogate marker for arginine vasopressin, which indicates neurohormonal activity. MR-proADM and MR-proANP reflects vascular tone and fluid balance. Among these biomarkers, GDF-15 stands out, demonstrating reliable prognostic value in both acute and chronic heart failure conditions, providing independent risk assessment and additional predictive power beyond NT-proBNP, troponin, and ST2 17, 24, 34.

Multimarker assessment may improve risk stratification because individual biomarkers represent different biological domains. Current ESC and AHA/ACC/HFSA guidance support natriuretic peptides for diagnosis and prognosis and recognizes cardiac troponin for myocardial injury assessment; however, GDF-15 is not currently recommended for routine heart-failure diagnosis, prognostic testing, or treatment guidance 1-3, 35.

## Molecular Biology of GDF-15

GDF-15 functions as a member of the TGF-β superfamily under its alternative names, a macrophage inhibitory cytokine-1 (MIC-1). GDF-15 is initially synthesized as a 40-kDa precursor protein that undergoes intracellular processing and dimerization to form a biologically active 25-kDa disulfide-linked dimer within the endoplasmic reticulum 9, 36. The level of GDF-15 is low under physiological conditions (typically ~200-1,200 ng/L); however, stress signals lead to substantial increases, making it a potential biomarker of disease 11, 18, 23.

The transcriptional activation of GDF-15 occurs in response to different cellular stress conditions. The stress response encompasses both oxidative and nitrosative stress, which activates p53 and NF-κB signaling pathways in conjunction with biomechanical signals from myocardial stretch and pressure overload, thereby activating AT1 receptors. Myocardial ischemia, accompanied by tissue inflammation and the proinflammatory cytokines TNF-α, IFN-γ, and IL-1β, activates GDF-15 expression 11.

GDF-15 is produced by multiple cell types and tissues, mainly when pathological conditions occur, presenting in cardiomyocytes, together with endothelial cells, atherosclerotic plaque smooth muscle cells, and activated macrophages during cardiovascular disorders 11, 18, 37. The protein is present throughout the cardiovascular system and also appears in adipose tissue, liver, kidneys, and other peripheral organs during systemic illness. The extensive distribution of GDF-15 suggests its function as a general cellular stress marker, rather than a specific indicator for particular organs or diseases 6, 7, 11.

## GDF-15 in Chronic Heart Failure

The medical use of GDF-15 as a biomarker for chronic heart failure (CHF) has been the subject of extensive research. Studies and trials over the last twenty years have shown that GDF-15 could potentially serve as an independent prognostic indicator surpassing traditional markers, including natriuretic peptides and troponins 7, 19, 23, 27.

GDF-15 levels in CHF patients show direct relationships with disease severity and functional class, and long-term mortality rates. The Valsartan Heart Failure Trial (Val-HeFT) demonstrated that elevated GDF-15 levels predicted higher mortality rates and hospitalization risks, without affecting NT-proBNP measurements 17. GDF-15 levels show correlations with NYHA functional class and reduced peak VO₂, as well as comorbid conditions, including renal dysfunction and anemia, indicating its ability to measure both cardiac health and total disease burden 7.

GDF-15 has been shown to exert benefits in patients with heart failure with preserved ejection fraction (HFpEF), exhibiting increased levels in HFpEF patients since this condition triggers inflammation, endothelial dysfunction, and metabolic stress, characteristic features of HFpEF 38. The prognostic value of GDF-15 for predicting long-term mortality and Heart Failure-Related hospitalization has been confirmed through large community-based cohorts and registries 7, 19, 23, 27, 39, 40.

## GDF-15 in Acute Heart Failure

Although extensive research pertinent to GDF-15 has been carried out in CHF, its role in AHF has only recently gained attention 18, 24, 27. The clinical syndrome of AHF is a complex condition that includes multiple systems, stress, and injuries that standard biomarkers fail to detect. GDF-15 shows promise as a biomarker in this context since it responds quickly and strongly to various stress signals, including inflammation, oxidative injury, and hemodynamic compromise 8, 11, 20, 27.

Multiple clinical investigations have started to determine how GDF-15 predicts outcomes for patients who need hospitalization due to acute heart failure 11, 22, 23, 25, 27, 37. GDF-15 levels measured at the start and over time have been shown to provide enhanced predictive information regarding mortality and rehospitalization rates, compared to NT-proBNP 21, 22, 24, 41. Beyond reflecting myocardial stress, GDF-15 is also upregulated in extra-cardiac tissues specifically the kidneys, liver, lungs, and adipose tissue under conditions of hypoxia, oxidative injury, and inflammation. This systemic expression enables GDF-15 to capture multi-organ dysfunction, particularly renal and hepatic impairment, decisive prognostic factors in acute heart failure. Consequently, GDF-15 provides incremental value for risk assessment, discharge planning, and guiding of closer post-discharge monitoring in AHF patients.

The translational evidence is organized in three tables. **Table 1** summarizes cellular and human-tissue studies of GDF-15 induction, secretion, signaling, and cytoprotection under biomechanical stretch, nitrosative stress, and simulated ischemia/reperfusion. **Table 2** summarizes *in vivo* ischemia/reperfusion and pressure-overload models, including the consequences of GDF-15 deficiency or overexpression on infarct size, hypertrophy, and ventricular function. **Table 3** summarizes clinical cohorts according to study design, sample size, sampling time, follow-up duration, outcome, and whether admission, discharge, or serial GDF-15 measurements were associated with mortality or heart-failure rehospitalization.

## Mechanistic Data from *in vitro* Models of Biomechanical Stress, Hypertrophy, and Ischemia/Reperfusion

Three complementary *in vitro* models have been reported. First, in NRVCMs subjected to biaxial stretch to 112% of baseline length for 24 hours, GDF-15 mRNA increased 24.8-fold, whereas phenylephrine and endothelin-1 produced no significant induction. Angiotensin II increased GDF-15 2.4-fold, and irbesartan reduced stretch-induced GDF-15 expression by 65.9%, supporting an AT1-receptor-dependent mechanotransduction pathway 8. Second, nitrosative stress induced by the nitric oxide donor SNAP increased GDF-15 mRNA approximately 17- to 28-fold in expression-array experiments and promoted secretion of mature GDF-15. During simulated ischemia/reperfusion, recombinant GDF-15 at 20 ng/mL reduced LDH release during ischemia and reduced apoptosis during reperfusion, as assessed by annexin V/propidium iodide flow cytometry, histone ELISA, and TUNEL staining. These effects were abolished by PI3K inhibitors or dominant-negative Akt1, establishing a PI3K/Akt-dependent survival mechanism 11. Third, adenoviral GDF-15 expression or recombinant GDF-15 attenuated phenylephrine/angiotensin II- or serum-induced increases in cardiomyocyte surface area and [3H]-leucine incorporation. GDF-15 activated SMAD2/3 and transiently activated Akt and ERK1/2; inhibitory SMAD6 or SMAD7 reversed its antihypertrophic effect 6. **Table 1** summarizes the experimental conditions and quantitative findings, while **Figure 1** integrates the upstream stressors, intracellular pathways, and phenotypic effects.

The models also demonstrate stimulus specificity. Although biaxial stretch increased GDF-15 approximately 24.8-fold, phenylephrine and endothelin-1 did not significantly alter expression, despite producing a similar approximately 1.9-fold increase in cardiomyocyte surface area 8. These findings indicate that GDF-15 induction is not simply a nonspecific consequence of cellular hypertrophy. The available evidence is nevertheless derived primarily from neonatal rodent cardiomyocytes, and the concentrations, timing, and mode of GDF-15 delivery differ across experiments.

*In vitro* research provides robust evidence showing that GDF-15 functions as a protective mediator responding to stress conditions. The protective actions of GDF-15 encompass traditional SMAD signaling pathways, and non-traditional PI3K/Akt signaling mechanisms, which regulate processes of hypertrophy, cell survival, and inflammation. Current evidence mainly originates from rodent neonatal cardiomyocytes. Future research should involve human-induced pluripotent stem cell (iPSC)-derived cardiomyocytes to validate mechanisms and identify cardiac-specific GDF-15 receptor complexes, also integrating stress-response biomarkers. Detailed therapeutic and biomarker potential for GDF-15 requires understanding of its upstream regulators and downstream effectors.

## Protective Roles of GDF-15 in *in vivo* Models of Ischemia/Reperfusion and Pressure Overload

*In vivo* studies support a protective role for endogenous GDF-15 (**Table 2**). In mice subjected to permanent or transient left anterior descending coronary artery ligation, myocardial GDF-15 mRNA increased within 1 hour, propeptide expression increased within 5 hours, and expression remained elevated for at least 7 days in the ischemic area 11. After 1 hour of ischemia followed by 24 hours of reperfusion, GDF-15-deficient mice had a comparable area at risk but a significantly larger infarct and approximately fourfold more TUNEL-positive cardiomyocytes in the border zone than wild-type mice 11. In a pressure-overload model, GDF-15 transgenic mice showed less hypertrophy after 2 weeks of transverse aortic constriction, whereas GDF-15-null mice showed greater heart-weight gain and early ventricular decompensation. In isolated working hearts, +dP/dt decreased by 84% in GDF-15-null mice after transverse aortic constriction compared with a 25% decrease in wild-type mice 6. Systemic adenoviral expression or twice-daily recombinant GDF-15 administration for 14 days also partially improved fractional shortening and ventricular dimensions in muscle-LIM-protein-deficient mice, supporting both autocrine/paracrine and endocrine actions 6.

Together, these *in vivo* studies confirm that GDF-15 is a stress-inducible cytokine that protects against both acute ischemic injury and chronic pressure overload by limiting infarct expansion, reducing cardiomyocyte loss, and attenuating pathological hypertrophy 6, 11. Despite this evidence, the precise pathways mediating these effects remain incompletely defined. For example, Kempf et al. suggested that GDF-15 modulates leukocyte infiltration into infarcted myocardia, although the specific immune mechanisms are not fully characterized 11. The absence of cardiac-specific knockout models also limits the ability to distinguish systemic from cardiomyocyte-specific effects.

Future investigations should employ tissue-specific knockout strategies and lineage-tracing approaches to clarify the cell-specific roles of GDF-15, while integrating *in vivo* data with *in vitro* mechanistic studies, potentially positioning GDF-15 not only as a biomarker but also as a therapeutic target in heart failure and related syndromes.

## Clinical Evidence Correlating GDF-15 with Outcomes

Clinical studies have evaluated GDF-15 using single admission measurements, discharge measurements, and serial trajectories **(Table 3)**. Across studies, median or mean concentrations were frequently several-fold higher than values reported in healthy populations, but the most informative sampling time differed among cohorts. The quantitative results below are presented to distinguish the prognostic contribution of baseline concentration from that of persistent elevation or an increasing trajectory.

### Short-term outcomes (< 1 year)

For short-term outcomes, Miftode et al. studied 173 participants and reported that GDF-15 was higher in patients with AHF than in ambulatory chronic-HF controls {596 [305-904] vs. 216 [139-305] ng/L} and independently predicted mortality 27. In the Thai prospective cohort by Kosum et al., median GDF-15 decreased from 6,346 pg/mL at admission to 5,711 pg/mL at discharge; admission GDF-15 was associated with 30-day mortality, while lower discharge GDF-15 and a greater in-hospital reduction were associated with lower 30-day rehospitalization risk 37. In RELAX-AHF (n=1,088 with available samples), baseline GDF-15 was not independently associated with 60-day heart-failure/renal-failure readmission or 180-day cardiovascular death, whereas larger increases at days 2 and 14 were associated with both outcomes 24. Similarly, in the FIGHT cohort, each 1,000-pg/mL increase in GDF-15 from baseline to 30 days was associated with a 35% higher risk of death or heart-failure hospitalization at 180 days (hazard ratio 1.35, 95% confidence interval 1.11-1.64) 25.

### Long-term outcomes (≥ 1 year)

For outcomes at 1 year or longer, admission GDF-15 was independently associated with adverse events in several cohorts. In 260 patients with AHF, GDF-15 predicted 1-year mortality with an area under the curve (AUC) of 0.707; combining GDF-15 with NT-proBNP increased the AUC to 0.743 26. Among 158 patients, discharge GDF-15 >=3,000 ng/mL was associated with a 1.86-fold adjusted risk of 2-year mortality, and simultaneous elevation of GDF-15 and BNP was associated with a 4.33-fold risk compared with both markers below their means 19. In 380 patients with acute HFpEF, the addition of GDF-15 increased the c-statistic from 0.643 to 0.657 for 1-year heart-failure readmission and from 0.638 to 0.660 for 1-year mortality; the highest GDF-15 tertile was associated with a hazard ratio of 2.25 for readmission 23. In the TRIUMPH cohort, serial GDF-15 independently predicted death or heart-failure rehospitalization after simultaneous modeling with NT-proBNP, ST2, galectin-3, troponin I, and creatinine (adjusted hazard ratio 1.44 per 1-SD increase in log GDF-15); a model combining GDF-15, NT-proBNP, and troponin I achieved an AUC of 0.785 22.

Not all studies support GDF-15 as a useful isolated measurement. In 104 patients studied by Plonka et al., GDF-15 alone at admission or discharge was not independently associated with the 1-year composite of death or heart-failure rehospitalization. However, the combined GDF-15/NT-proBNP model measured at the 30-day visit achieved an AUC of 0.75, outperforming either biomarker alone 41. These data reinforce two themes illustrated in Figure 1: GDF-15 reflects a broad stress response rather than congestion alone, and its greatest clinical value may lie in serial and multimarker assessment. Human autopsy tissue further supports biological plausibility: GDF-15 propeptide expression was approximately four- to fivefold higher in infarcted myocardium than in noncardiac controls and remained elevated from less than 12 hours to 14 days after symptom onset 11.

The clinical evidence indicates that GDF-15 has the potential to serve as a significant biomarker for risk assessment in patients with acute heart failure. Future research should focus on standardizing the clinical thresholds of GDF-15, demonstrating its added value through extensive observational studies, and evaluating whether GDF-15-based therapeutic management protocols can lead to improved patient outcomes within heart failure treatment plans.

## Limitations of GDF-15

Several limitations currently prevent routine clinical application. First, GDF-15 is not cardiac-specific and may be increased in cancer, chronic kidney disease, pulmonary or hepatic injury, inflammation, aging, and metabolic disease 10, 12-16. Second, assay platforms, sampling schedules, transformations, and proposed cutoffs vary substantially; reported thresholds include approximately 3,000-5,115 pg/mL, which cannot yet be transferred reliably between populations 19, 21, 26, 41. Third, most evidence is observational, and associations may reflect residual confounding by age, renal dysfunction, anemia, frailty, or multiorgan disease. Fourth, single measurements may miss clinically relevant trajectories, as demonstrated by RELAX-AHF, FIGHT, and TRIUMPH 22, 24, 25. Finally, no randomized trial has established that GDF-15-guided treatment decisions improve patient outcomes.

## Clinical Implications and Current Guideline Recommendations

GDF-15 can be effective as an assessment tool supplementary to conventional methods. The prognostic value of GDF-15 has been demonstrated across various patient groups in multiple healthcare settings, operating independently of NT-proBNP and natriuretic peptides. The extensive biological processes measured by GDF-15, including inflammation, oxidative stress, and cellular injury, make it an excellent prognostic tool beyond conventional hemodynamic stress markers.

The available data suggest that GDF-15 is best considered a complementary prognostic biomarker. Persistent elevation at discharge, an increase during hospitalization or early follow-up, and concurrent elevation with NT-proBNP identify particularly high-risk groups. However, these associations should not yet be used as stand-alone criteria for discharge, treatment escalation, or follow-up intensity outside research settings.

International heart-failure guidelines and consensus pathways do not currently recommend GDF-15 for routine diagnosis, prognostic testing, or treatment guidance. The 2021 ESC guideline and 2023 focused update emphasize natriuretic peptides, while the 2022 AHA/ACC/HFSA guideline and 2024 ACC pathway incorporate natriuretic peptides and cardiac troponin within clinical assessment 1-3, 35. Thus, the clinical role proposed for GDF-15 in this review is investigational and depends on assay standardization, external validation of thresholds, and prospective demonstration of clinical utility.

The management of heart failure in the future could benefit from integrating GDF-15 as a clinical tool. The risk stratification process benefits from GDF-15 as a high-risk patient identification tool in HFpEF patients and those with multi-organ dysfunction and persistent systemic inflammation, as risk stratification is shifting toward more personalized approaches. The development of standardized cut-off values, rapid point-of-care tests, and automated risk prediction models that utilize GDF-15 data will increase its efficacy as part of a comprehensive biomarker panel during hospital discharge, early outpatient visits, and treatment response assessments. Future clinical trials should evaluate GDF-15-guided strategies to determine whether this biomarker can be used to transition from a prognostic tool to a therapeutic decision-making aid, thereby improving patient outcomes.

## Conclusion

GDF-15 links cellular stress biology with prognosis in AHF. In experimental models, biomechanical stretch increased GDF-15 approximately 24.8-fold; recombinant GDF-15 reduced ischemia/reperfusion-related cell death through PI3K/Akt signaling and attenuated hypertrophy through SMAD2/3-associated pathways. GDF-15 deficiency increased infarct size and apoptosis after ischemia/reperfusion and produced marked ventricular decompensation during pressure overload. In clinical cohorts, admission and discharge concentrations and, particularly, serial increases were associated with mortality or heart-failure rehospitalization, with incremental performance when GDF-15 was combined with NT-proBNP and troponin. Nevertheless, GDF-15 remains a nonspecific prognostic marker without standardized assays, validated universal thresholds, or evidence that biomarker-guided management improves outcomes. Future work should prioritize assay harmonization, prespecified serial-sampling protocols, external validation across AHF phenotypes, and randomized studies of GDF-15-informed care.

## Figures and Tables

**Figure 1:**
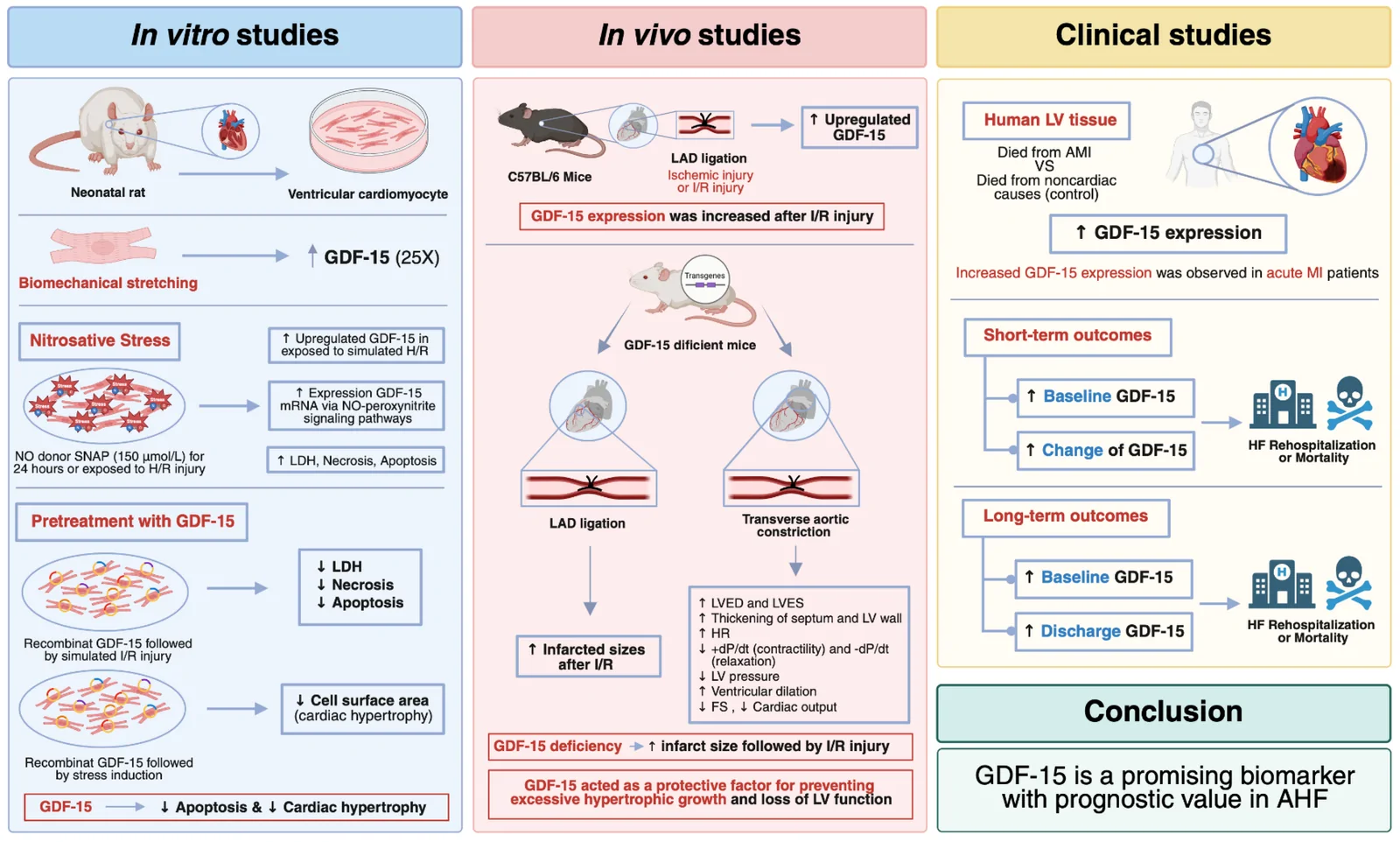
Integrated mechanistic and clinical roles of GDF-15 in acute heart failure. Biomechanical stretch, ischemia/reperfusion, oxidative and nitrosative stress, and inflammatory signaling induce GDF-15 expression in cardiomyocytes and extracardiac tissues. Experimental studies indicate that GDF-15 activates SMAD2/3, PI3K/Akt, and ERK1/2 pathways, thereby reducing hypertrophy, necrosis, and apoptosis. *In vivo*, GDF-15 deficiency increases infarct size, border-zone apoptosis, pressure-overload hypertrophy, and ventricular dysfunction. Clinically, higher admission or discharge concentrations and increasing serial GDF-15 levels are associated with mortality and heart-failure rehospitalization, particularly when combined with NT-proBNP or troponin. The figure also emphasizes the principal barriers to clinical implementation: lack of cardiac specificity, assay heterogeneity, nonuniform thresholds, and absence of interventional evidence. (Created in BioRender. Kosum, P. [2026] https://BioRender.com/2wr6t5u).

**Table 1:** GDF-15 expression under cardiac stress and its effects on cardiomyocytes: Reports from *in vitro* studies and clinical samples.

Model	Intervention (drug/duration)	Major findings				Interpretation	References
		Cellular metabolism	Neurohumoral and immune responses	Apoptosis/ oxidative stress /survival study	Cardiac parameters		
Neonatal rat ventricular cardiomyocytes underwent * Biomechanical stretching: Biaxial stretching (purified NRVCMs up to 112% of baseline length for 24 hours * Pharmacological stretching: phenylephrine (50 µmol/L) *Unstretched cardiomyocyte		↑ GDF-15 (24.8x) ⇿ GDF-15 ⇿ GDF-15	↑ Heme oxygenase-1 and metallothionein-1 ↑ Induction mediated via AT1 receptor			GDF-15 is a mechanosensitive cytokine that is induced by mechanical stretch through the activation of the AT1 receptors.	(8)
Neonatal ventricular cardiomyocytes underwent nitrosative stress (NO donor SNAP (150 µmol/L) for 24 hours or exposed to H/R injury		↑ Upregulated GDF-15 in exposed to simulated I/R ↑ Expression GDF-15 mRNA via NO-peroxynitrite signaling pathways ↑ PI3K- and Akt- dependent mechanism	↑ IL-1β ↑ IFN-γ ↑ NOS2 activation	↑ LDH ↑ Necrosis ↑ Apoptosis		GDF-15 expression was increased following H/R injury via NO-peroxynitrite signaling pathways, and recombinant GDF-15 treatment attenuated apoptosis in simulated H/R injury.	(11)
	Pretreatment with recombinant GDF-15 followed by simulated ischemia for 3 hours followed by reperfusion for 1 hour			↓ LDH ↓ Necrosis ↓ Apoptosis			
Neonatal cardiomyocytes underwent stress induction via combination of PE (50 µmol/L)/Ang II (1 µmol/L), or 1% FBS for 48 hours	Pretreatment with recombinant GDF-15 adenovirus	↑ Activated SMAD2/3 pathways ↑ Activated Akt and ERK1/2 pathways	↓ [^3^H] Leucine		↓ Cell surface area (cardiac hypertrophy)	GDF-15 treatment attenuated cardiomyocyte hypertrophy through SMAD2/3/Akt/ERK pathways.	(6)
Human LV tissue: died from AMI VS died from noncardiac causes (control)		↑ GDF-15 expression				Increased GDF-15 expression was observed in acute MI patients.	(11)

**Abbreviations:** I/R, ischemic/reperfusion; NO, nitric oxide; IL-1β, interleukin 1 beta; IFN-γ, interferon gamma; NOS, nitric oxide synthetase 2; AT1, angiotensin 1; NRVCMs, neonatal rat ventricular cardiomyocytes; PI3K, phosphoinositide; 3-OH kinase SNAP, S-nitroso-N-acetyl-D,L-penicillamine; Ang II, Angiotensin 2; PE, phenylephrine; mRNA, messenger ribonucleic acid, ERK, extracellular signal-regulated kinases; LDH, lactate dehydrogenase; [3H] Leucine, Tritiated Leucine; H/R, hypoxia/reoxygenation.

**Table 2:** The effects of Growth Differentiation Factor 15 (GDF-15) on cardiac parameters: Reports from *in vivo* studies.

Model	Major findings			Interpretation	References
	Cellular metabolism	Apoptosis/oxidative stress/survival study	Cardiac parameters		
C57BL/6 Mice underwent Ischemic injury or I/R injury: transient LAD ligation for 1 hour followed by reperfusion or permanent LAD ligation	↑ Upregulated GDF-15 in exposed to simulated I/R			GDF-15 expression was increased after I/R injury, whereas GDF-15 deficiency resulted in increased infarct size following I/R injury.	(11)
GDF-15 deficient mice underwent transient LAD ligation (for 1 hour followed by reperfusion for 24 hours)		↑ Infarcted size after I/R			
GDF-15 deficient mice underwent transverse aortic constriction (TAC): for 2 weeks			↑ LVED and LVES ↑ Thickening of septum and LV wall ↑ HR ↓ +dP/dt (contractility) and -dP/dt (relaxation) ↓ LV pressure ↑ Ventricular dilation ↓ FS , ↓ Cardiac output	GDF-15 acted as a protective factor for preventing excessive hypertrophic growth and loss of ventricular function.	(6)

**Abbreviations:** I/R, ischemic/reperfusion; LV, left ventricle; LVED, left ventricular end diastole; LVES, left ventricular end systole; HR, heart rate, dP/dt; ratio of delta pressure and time; LAD, left anterior descending artery; HF, heart failure; WT, wild-type.

**Table 3:** The correlation of Growth Differentiation Factor 15 (GDF-15) with clinical outcomes in acute heart failure: Reports from clinical studies.

Study type	Sample size (N)	Follow up time	Primary outcome	Major findings (based on GDF-15 during admission, discharge, and change)						Interpretation	References
				Rehospitalization or mortality							
				Short term (< 1 year)			Long term (≥ 1 year)				
				Admission	Discharge	Change	Admission	Discharge	Change		
Prospective	1,088	6 months	60-day HHF/RF or CV mortality and 180-day CV mortality	0	N/A	+ (H/M)				Increase in changes of GDF-15 level was correlated with higher short-term rehospitalization or CV mortality.	(24)
	107	1 year	1-year all-cause mortality				+ (H/M)	N/A	N/A	Higher baseline GDF-15 correlated with higher long-term rehospitalization or all-cause mortality.	(18)
	158	2 years	2-year all-cause mortality				N/A	+ (M)	N/A	Higher discharge GDF-15 correlated with higher long-term all-cause mortality.	(19)
	260	1 year	1-year all-cause mortality				+ (M)	N/A	N/A	Higher baseline GDF-15 correlated with higher long-term all-cause mortality.	(26)
	830	1 year	1-year all-cause mortality				+ (M)	+ (M)	N/A	Higher baseline and discharge GDF-15 correlated with higher long-term all-cause mortality.	(20)
	380	2 years	2-year all-cause mortality and CV mortality				+ (M)	0	0	Multiple baseline biomarkers (induced GDF-15) higher with higher long-term all-cause or CV mortality.	(31)
	249	6 months	180-day HHF or all-cause mortality	0	N/A	+ (H/M)				Increase in changes of GDF-15 level over 30 days correlated with short-term rehospitalization or all-cause mortality.	(25)
	173	30 days	30-day all-cause mortality	+ (M)	N/A	N/A				Higher baseline GDF-15 correlated with higher short-term all-cause mortality.	(27)
	475	1 year	1-year HHF or all-cause mortality				+ (H/M)	N/A	N/A	Higher baseline GDF-15 correlated with higher long-term HHF or all-cause mortality.	(22)
	380	1 year	1-year all-cause mortality				+ (M)	N/A	N/A	Higher baseline GDF-15 correlated with higher long-term HHF or all-cause mortality.	(23)
	92	6 months	30-day HHF or 30-day all-cause mortality	0/+ (H/M)	0	0				Higher baseline GDF-15 correlated with higher short-term all-cause mortality.	(37)
	104	1 year	1-year HHF or all-cause mortality				0	0	N/A	Higher GDF-15 alone not associated with long-term HHF or all-cause mortality.	(41)
Retro- spective	249	3 years	1-year and 3-year all-cause mortality				+ (M)	+ (M)	0	Higher baseline and discharge GDF-15 correlated with higher long-term all-cause mortality.	(21)

**Abbreviations:** RF, renal failure; CV, cardiovascular; HHF, heart failure rehospitalization; Change, absolute reduction of GDF-15 level from admission to discharge; Mortality, defined as all-cause or cardiovascular mortality; H, rehospitalization; M, mortality; H/M, rehospitalization or mortality; +, positive correlation; 0, no correlation; N/A, not available.

## Abbreviations

AHF: acute heart failure
GDF-15: growth differentiation factor-15
HF: heart failure
NT-proBNP: N-terminal pro-B-type natriuretic peptide
BNP: B-type natriuretic peptide
HFpEF: heart failure with preserved ejection fraction
NRVCMs: neonatal rat ventricular cardiomyocytes
TAC: transverse aortic constriction
I/R: ischemia/reperfusion
